# ExpressionPlot: a web-based framework for analysis of RNA-Seq and microarray gene expression data

**DOI:** 10.1186/gb-2011-12-7-r69

**Published:** 2011-07-28

**Authors:** Brad A Friedman, Tom Maniatis

**Affiliations:** 1 Department of Molecular and Cell Biology, Harvard University, 7 Divinity Ave, Cambridge, MA 02138, USA; 2 The Koch Institute for Integrative Cancer Research, Massachusetts Institute of Technology, Cambridge, MA 02139, USA; 3 Department of Bioinformatics and Computational Biology, Genentech, Inc., 1 DNA Way, South San Francisco, CA 94080, USA; 4 Department of Biochemistry and Molecular Biophysics, Columbia University College of Physicians and Surgeons, 701 West 168th St, New York, NY 10032, USA

## Abstract

RNA-Seq and microarray platforms have emerged as important tools for detecting changes in gene expression and RNA processing in biological samples. We present ExpressionPlot, a software package consisting of a default back end, which prepares raw sequencing or Affymetrix microarray data, and a web-based front end, which offers a biologically centered interface to browse, visualize, and compare different data sets. Download and installation instructions, a user's manual, discussion group, and a prototype are available at http://expressionplot.com/.

## Rationale

RNA-Seq has emerged in recent years as the eminent platform for analysis of gene expression and RNA processing [1-3]. However, processing the raw sequence data to get useful and accurate information about gene expression and RNA processing is still a daunting task, even for computationally inclined researchers. High quality software packages now exist to perform specific steps in the analysis pipeline [4-10], as well as web-based systems such as Galaxy [11] and GenePattern [12] that enable the management of data flow through these tools. We present ExpressionPlot, an open source solution consisting of a back end pipeline, which performs alignment and statistical analyses, and a web-based front end, which allows users to explore and further compare the completed analyses. Compared to Galaxy and GenePattern, ExpressionPlot's web-based front end is novel in the ease with which one can browse and manipulate gene expression results: gene/isoform lists are one-click filterable, sortable and hyperlinked to the underlying genomic regions in the table_browser tool. Furthermore, even with differing platforms (such as microarray versus RNA-Seq) or organisms (such as mouse versus human), the front end can automatically compare changes in gene expression across different experiments using the 4 way and heatmap tools.

ExpressionPlot can be tested as a virtual machine (running under VirtualBox), or installed directly into an existing web server. Input to ExpressionPlot can be raw sequence data (FASTQ files) or Affymetrix array data (CEL files), completed alignments (BAM files), or tables of gene expression values and changes generated by other back ends. Once data are pre-processed, the web-based front end allows users to easily browse measures of quality control, plot changes in gene expression and RNA processing, browse hyperlinked tables of changed genes and splicing events, generate read plots from a genomic view, compare different datasets (including from different organisms or between microarray and RNA-Seq), generate empirical cumulative distribution functions (ECDFs) to look at levels or changes in a cohort of genes, and look up levels of specific genes.

The ExpressionPlot back end can also generate BAM and BigWig files upon request, and for downstream analysis the web-based front end can output spreadsheets with gene and exon statistics. ExpressionPlot includes a web-controllable user account and access control system by which pre-published data can be shared with other users, or, when appropriate, made public. Finally, ExpressionPlot does not require a cluster; it can run on any machine with sufficient memory to hold the bowtie indexes (usually at least 3 or 4 GB) and hard drive space to hold the sequencing data and processed files (roughly 1 to 2 GB per lane).

In short, ExpressionPlot is a unified solution for gene expression analysis of RNA-Seq and microarray data.

## Tasks of gene expression analysis

RNA-Seq and microarray analyses begin with the following pre-processing tasks. Back end pre-processing tasks (RNA-Seq): 1, alignment; 2, read accumulation; 3, statistical calculations. Back end pre-processing tasks (microarrays): 1, background subtraction; 2, probe normalization; 3, probe accumulation; 4, statistical calculations.

The pre-processing tasks are sequential and usually performed for all analysis projects. In ExpressionPlot they are performed by the back end, which is started from the command line on the server. A typical RNA-Seq data set might take a few days to run, most of which is spent on alignments. Using pre-aligned data sets is possible by importing from BAM files.

Once the pre-processing tasks have been completed, the subsequent tasks can be considered a mixture of global (discovery-based) and specific (hypothesis-based) tasks. In ExpressionPlot these tasks are the domain of the web-based front end, and all run on-demand within seconds. Global tasks: (a) quality control; (b) generation of plots and tables of changed genes/events; (c) genome-wide comparison of changes from different experiments/data sets. Specific tasks: (a) examining reads/probe intensities from a particular genomic region; (b) examining levels/changes of a particular gene/splicing event or set of genes/splicing events.

ExpressionPlot provides simple mechanisms to perform all of these steps.

### Back end pre-processing tasks (RNA-Seq)

#### Alignment

ExpressionPlot uses bowtie [9] to align reads to the genome and then a database of splice junctions. The splice junction databases that come with ExpressionPlot were generated by combining the known half-junctions from each gene in every possible forward-splicing combination (exon *n *splices to exon *m *where *m *>*n*). Pre-computed junction databases can be downloaded and installed with the EP-manage.pl script (human, mouse and rat as of press time) or can easily be generated using the make_junctions_database.pl script that comes with ExpressionPlot. ExpressionPlot's alignment strategy is to find and use only unique best alignments either to the genome or to the splice junction database (Figure S1 in Additional file 1). For paired-end data an additional step is taken to try to align the single ends individually (Figure S2 in Additional file 1).

#### Counting reads for genes and RNA processing events

Aligned reads are then mapped to gene models and alternative splicing events. Users can supply their own models and events or download and install pre-computed annotations using EP-manage.pl (currently available for human, mouse and rat). The pre-computed gene models are built from all exons of any transcript (based on UCSC known genes [13] or Ensembl [14]). A read is counted towards any gene that contains the aligned positions, possibly split by a junction, on either strand within its exons. Scripts and detailed instructions to generate annotations for other genomes are included.

Pre-computed candidate skipped exon events are created from all known exons, regardless of whether or not they are known to be skipped. For skipped exons, skipping reads are considered as splice junction-spanning reads that both skip the exon and are additionally anchored in known splice sites of the host genes (Figure S3 in Additional file 1).

For intron retention, the number of reads aligning to the intron is compared to the number aligning to locally constitutive flanking exons (Figure S4 in Additional file 1). Locally constitutive means that, based on the underlying annotation, all transcripts flanking that intron contain those exons (Figure S5 in Additional file 1). As with skipped exons, the pre-computed sets contain candidate events for all known introns.

Finally, alternative terminal exon events are created for genes with multiple transcript start sites (TSSs) or multiple poly-adenylation/cleavage sites (PACS). These events compare reads supporting a candidate terminal exon with more distal (5' of TSS or 3' of PACS) exons. Such events are created for all but the 5'-most TSS and 3'-most PACS (Figure S6 in Additional file 1).

Support for other types of events, including alternative splice sites and sequence variants (due to single nucleotide polymorphisms or RNA editing), is planned for a future release.

#### Statistical calculations

For changes in gene expression ExpressionPlot uses the DESeq package [15] to model biological variation in the calculation of *P-*values. This package normalizes samples using median fold change, and models the read counts using the negative binomial distribution, including a term for both sampling and biological noise. Alternatively, users can choose a modification of a previously described procedure [16] to detect technical differences between two lanes or groups of lanes. In a similar spirit to DESeq and other existing packages [17,18], total read counts are normalized using a robust procedure that is not dominated by the mostly highly expressed genes. In this step, the effective total number of reads in each sample is optimized to minimize the resultant number of significantly changed genes, a procedure we call 'minimize significant changes' (Methods, Supplementary Methods in Additional file 1 and example data in Additional file 2). Finally, a binomial test is performed on the number of reads aligning to a particular gene from the two samples to determine if the ratio is significantly different from the ratio of total numbers of reads in the two samples (Supplemental Methods in Additional file 1).

For the RNA processing events, we form two-by-two contingency tables looking at the numbers of reads supporting the two isoforms in the different samples (for example, Figures S3, S4, and S6 and Supplementary Methods in Additional file 1). The *P*-values are then derived from either Fisher's exact test (which is known to be conservative in this regime (Supplementary Methods in Additional file 1) or, if all the 'expected values' are greater than 5, the Chi-squared test.

By default, the ExpressionPlot back end generates *P*-values that are not adjusted for multiple testing. This should be kept in mind when setting cutoffs on the website. We usually use a *P*-value cutoff of 10 ^4 ^. For example, using the UCSC genes cluster for mouse (mm9) there are 27,389 genes, so, on average, this cutoff would yield no more than 3 false positives. Actually, in most RNA-Seq data sets many of the genes are not expressed or are expressed at extremely low levels, and so the expected number of false positives is even lower since the small *P*-values are not achievable for these genes. Users who prefer to work with Benjamini-Hochberg-corrected *P*-values can choose to do so by providing the correct switches as described in the User's Guide.

### Pre-processing tasks (microarrays)

#### Background subtraction and probe normalization

ExpressionPlot uses Affymetrix Power Tools [19] to perform the background subtraction using either mismatch probes (3' UTR arrays) or GC-control probes (exon arrays), and follows this with quantile normalization of background-subtracted probe intensities. Users can use any affymetrix array for which they have the appropriate library files, but for the following arrays those files can be automatically downloaded and installed by EP-manage.pl: HG-U133 (A/B), HG-U133_Plus_2, HuExon, MOE430 (A/B), MoExon and Rat230_2.

#### Statistical calculations

For microarray data, gene levels are estimated first by finding all 'detected probes', which are defined as probes with positive (background-subtracted) intensities across all arrays in the project. Once these probes are defined, the gene level in each array is summarized as the median probe intensity. *P *values for gene level changes are calculated by default using the Limma package [20], or, optionally, the *t*-test. As with the RNA-Seq pipeline, the *P*-values are not by corrected for multiple testing unless specifically requested.

### Web-based front end: global tasks

**Figure 1:**
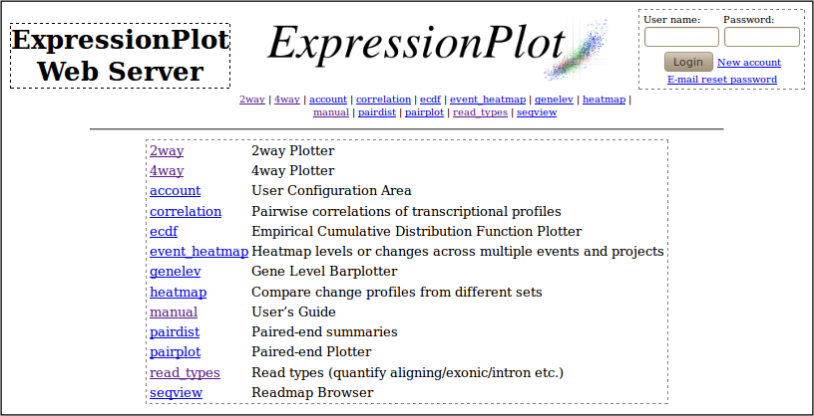
**The ExpressionPlot home page**. The website opens with this screen, giving a list of tools available in ExpressionPlot, and a login box in the top right. The navigation bar on top appears on all pages, giving links to the other tools. The 'manual' link is context-aware: it automatically opens the User's Guide (in another tab) to the page explaining the current tool.

Website users are initially presented with a landing page with links and short descriptions of all the different tools available in ExpressionPlot (Figure 1). The navigation bar at the top, as well as the login box on the top right, are present on every page during the website experience for easy navigation. The 'manual' link opens the page of the User's Guide relevant to the currently selected tool.

#### Quality control

**Figure 2:**
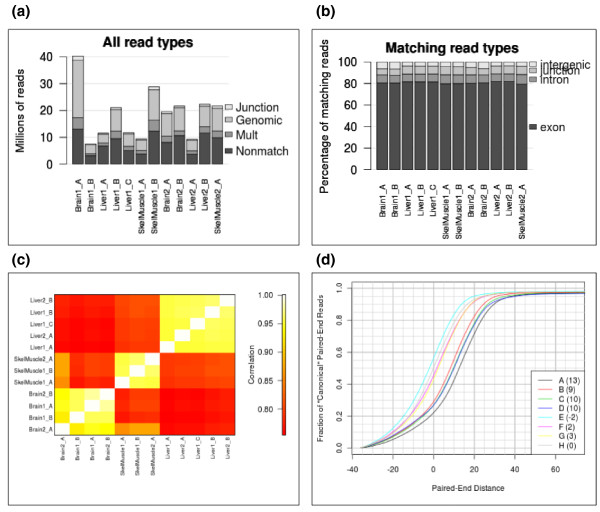
**Screen shots of ExpressionPlot quality control tools**. **(a) **read_types tool showing all read types. Numbers of non-aligning (Nonmatch), mulitply-aligning (Mult), unique genome-aligning (Genomic) and unique junction-aligning (Junction) reads are shown for each lane from a mouse tissue transcriptome dataset [3]. Numbers (1/2) indicate different libraries; letters (A/B/C) indicate different lanes of the same library. **(b) **read_types tool showing matching read types, normalized to 100%. **(c) **Pairwise correlation heatmap of gene expression profiles generated from each lane. **(d) **pairdist tool shows ECDF of paired-end distances of 'canonical' reads (same chromosome, different strand, minus strand read downstream of plus strand read). 'Distance' is defined as the genomic distance, in nucleotides, between the aligned positions of the last sequenced bases of the two reads (can be negative if the alignments overlap). The samples have been de-identified (data in Additional file 3). Numbers in parentheses indicate median paired-end distance for each sample (add 36 for both sequences and 50 for both Illumina adaptors (+172) to get complete library size).

The ExpressionPlot front end provides several quality control tools for RNA-Seq data. The read_types tool graphs the number of reads in each sample of each 'type': non-aligning, multiply-aligning, paired-end uniquely aligning, or single-end uniquely aligning (Figure 2a). The user can also run this tool looking at only the uniquely aligning reads to see if they align to exons, introns, intergenic regions or junctions (Figure 2b). The correlation tool generates either a heatmap or a hierarchical clustering dendrogram showing the pairwise correlations of gene expression profiles in the RNA-Seq or microarray samples of your project (Figure 2c; Supplementary Methods in Additional file 1).

For paired-end data sets, the pairdist tool shows the fraction of paired end reads for which (1) the two ends align to different chromosomes, (2) the two ends align to the same chromosome but on the same strand, (3) the two ends align to the same chromosome and different strands but the minus end strand is upstream of the plus end strand, and (4) the two ends align to the same chromosome, different strands, minus end downstream of the plus end but there is at least one intron between the two ends. The fifth category of reads, where the two ends do not flank any known intron, can be used to estimate the insert size, and ECDFs of the insert sizes (defined as the length of the un-sequenced part of the library between the paired ends) for the different lanes are also plotted by this tool (Figure 2d; data in Additional file 3).

#### Generation of plots and tables of changed genes/events

**Figure 3:**
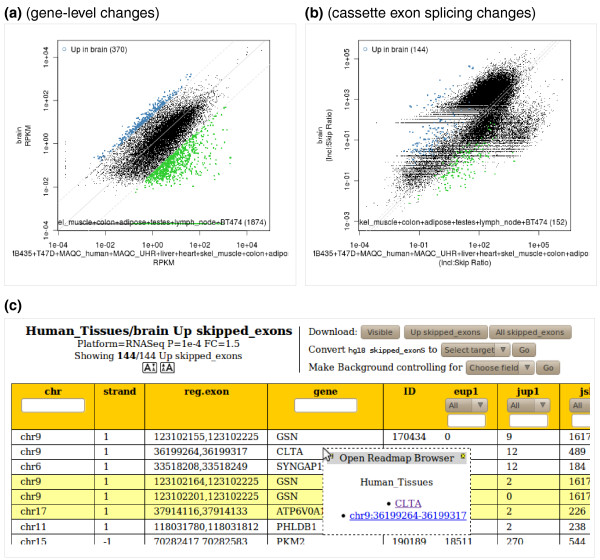
**Screen shots of ExpressionPlot 2way plot and table_browser**. **(a) **2way plot of human tissue panel RNA-Seq data [1] showing brain gene expression on the y-axis and average expression in all other tissues (pooled) on the x-axis. Blue points correspond to genes significantly higher (*P *≤ 10^-4^, fold change ≥20, 370 points) in brain relative to the other tissues; green points correspond to significantly lower. **(b) **2way plot showing cassette exon usage (inclusion:skip read ratios) instead of gene levels in the same data set. The heavy lobe above the diagonal corresponds to exons with zero skipping reads in the brain, and the lighter lobe below the diagonal corresponds to exons with zero skipping reads in all other tissues. Although the *P*-values are still valid, in these regimes the inclusion:skip ratio statistic is less precise. **(c) **Partial screen shot of table browser showing brain-enriched cassette exons in the same data set. The context menu was triggered by the mouse clicking on the row for *CLTA *(clathrin, light chain A) and offers the user links to open the seqview genome browser tool in a window covering either the entire gene or just the alternative exon. In either case the exon will be automatically highlighted (Figure 5).

The 2way tool and its associated table browser are the basic tools to examine the relationships between gene levels (or RNA processing events) in two different samples. The x-axis will correspond to one sample (such as 'wild type'), and the y-axis to another (such as 'mutant'). The project and pair of samples are chosen by the user from drop-down menus and the plots, like all the other plots in ExpressionPlot, are generated on demand by the web server. The 2way plot is a scattergram where points correspond to genes (or RNA processing events, for example, cassette exons), and are colored according to whether they are significantly different in the two samples (Figure 3a,b). *P*-value and fold-change cutoffs for significance can be controlled by the user.

After the plot is generated, action buttons are presented to the user to access the significantly changed genes or RNA processing events in the table browser. This screen presents the user with a dynamic table whose rows correspond to changed genes/events (Figure 3c). The columns of the table contain identifiers for the gene or event (like gene name, chromsome, strand and position), as well as all the associated statistics (such as read numbers, RPKM values (reads per kilobase gene model per million total reads), and *P*-values). The table can be sorted by clicking on the header of the desired field, or filtered using a text string or a numeric filter. Action buttons allow for the export of the table into other software, such as R or OpenOffice (or Excel), for automatic conversion of the genes into other IDs (such as Ensembl or Entrez), and for the automatic generation of expression-controlled background sets of similarly expressed but unchanged genes (in terms of either RPKM or raw read numbers - the user chooses, although we recommend raw read numbers to avoid transcript length biases [21]). These background sets are appropriate for downstream gene ontology or motif analysis.

A convenient feature of the table browser is the ability to click on any row to be presented with a link to the ExpressionPlot genome browser seqview. This browser displays both RNA-Seq reads, including those spanning junctions, as well as array probe intensities, along with gene annotations (described below).

#### Comparison of changes from different experiments/data sets

**Figure 4:**
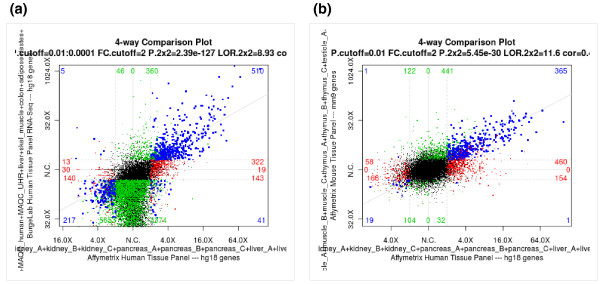
**Screen shots of ExpressionPlot 4way plots showing cross-platform and cross-species comparisons**. **(a) **Heart-enriched gene expression in human tissue panel exon array [28] (x-axis) and RNA-Seq [1] (y-axis) data sets. Points correspond to genes. Fold-change of expression in heart is plotted versus all other samples in corresponding data set. Genes enriched in heart are plotted further to the right (exon array) and/or up (RNA-Seq), and those higher in other samples are further to the left and/or down. Genes significantly different only on one platform are colored red (exon array) or green (RNA-Seq) and those different on both platforms are colored blue. *P*-value cutoffs are 0.01 for exon array and 10^-4 ^for RNA-Seq, and fold-change cutoffs are 2 for both platforms. Colored numbers show number of genes in each category. **(b) **Similar plot comparing the same x-axis (human heart-enriched gene expression by exon array) to mouse heart-enriched gene expression, also by exon array (y-axis).

Having examined changes in two different conditions of a single experiment, it is natural to ask how these changes compare to another experiment. Sometimes this second experiment may be part of the same project, but in other cases it could be part of another project, and maybe even have been performed on another platform (for example, RNA-Seq versus microarray) or in another organism (for example, human versus mouse). The 4way tool and its associated table browser automatically match up changed genes or RNA processing events from different experiments, presenting them in a similar manner to its 2way cousin. After selecting two projects, and a pairwise comparison, *P*-value and fold-change cutoff for each, ExpressionPlot generates a scattergram where each point corresponds to a gene (or event). Here the x-axis shows the change in that gene/event in the first comparison and the y-axis shows the change in the second comparison (Figure 4). For example, points in the upper right quadrant would correspond to genes/events increased in both experiments, whereas those in the upper left quadrant would be decreased in the x-axis experiment, but increased in the y-axis experiment. Points are colored according to whether the gene/event is significantly changed in one or both experiments, with blue representing those changed in both experiments.

As with the 2way tool, after the plot is generated ExpressionPlot offers the user action buttons to select a group of genes/events to further examine in the 4way table browser. For example, clicking 'Up/Up' would show a table of genes/events increased in both experiments. This table shows the annotation of the gene/event (identifier, chromosome, position, strand, and so on) as well as all the associated statistics. It has the same fields that would be shown in the 2way browser, but they are then repeated for both experiments. This includes the annotation fields, since sometimes they are from different organisms. As with the 2way browser, there are action buttons to download, convert IDs and generate background sets. Finally, clicking on a row of the table opens a context menu with links that will automatically open the genome browser to the right part of the genome for the two experiments. In the case of RNA processing events the correct genomic region will be automatically highlighted within the browser, so the user can quickly find, for example, a differentially spliced cassette exon.

The heatmap tool (Figure S8 in Additional file 1) allows the user to compare larger numbers of change profiles. Here all the different comparisons from one project are laid out along the x-axis and all the comparisons from a second (possibly different) project are laid out along the y-axis. The color of each square of the heatmap indicates the similarity of the two comparisons. The user can choose from a variety of statistics to quantify similarity. This tool is a useful way to look for relationships within larger numbers of experiments.

### Web-based front end: specific tasks

#### Examining reads from a particular genomic region

**Figure 5:**
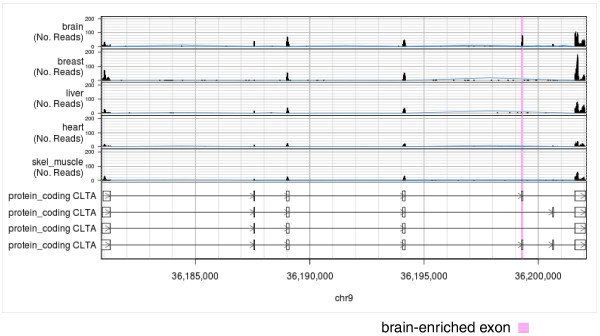
**Screen shot of ExpressionPlot's genome browser seqview**. The region of the *CLTA *gene, which contains a brain-enriched exon (pink), is shown. Known transcripts of *CLTA *are seen along the bottom (arrowheads indicate plus strand). The accumulation of RNA-Seq reads from five human tissues is shown on the top. The heights of black bars indicate numbers of reads overlapping each genomic position, whereas the heights of blue brackets indicate numbers of reads overlapping splice junctions. Data from RNA-Seq human tissue panel [1].

The seqview tool is ExpressionPlot*'s *genome browser (Figure 5). With it, the user can select the project of interest, then query either by a gene name or genomic region. One of several annotations can be chosen, and then a plot is generated showing either the pileup of reads in that region (with strands separated or merged, as requested by the user) or of the hybridization intensities of microarray probes in that region. Zooming and scrolling is implemented, and users can also highlight specific genomic coordinates. Barplots are automatically generated showing levels of genes within the requested regions.

The pairplot tool is a genome browser specifically designed to visualize the relationship between the aligned positions of paired ends. Only one sample can be visualized at a time. The gene annotation of the requested region is shown, as well as the pileup track from the seqview tool showing total numbers of reads. Above this a scattergram shows a point for each paired-end read aligning to the genomic region. The x-axis gives the position of the plus-strand end and the y-axis gives the position of the minus-strand end. The colors and sizes of the points indicate the number of reads aligning to each pair of coordinates. Under conditions of constitutive splicing, the scattergram should form a series of segments above each exon and parallel to the diagonal, with the distance to the diagonal dictated by the paired-end insert and intron size. Alternatively spliced regions, however, will show multiple parallel segments corresponding to the different isoforms. The relative strength of the segments corresponds to the abundances of the two isoforms (Figure S9 in Additional file 1).

#### Examining levels or changes of particular genes or events

**Figure 6:**
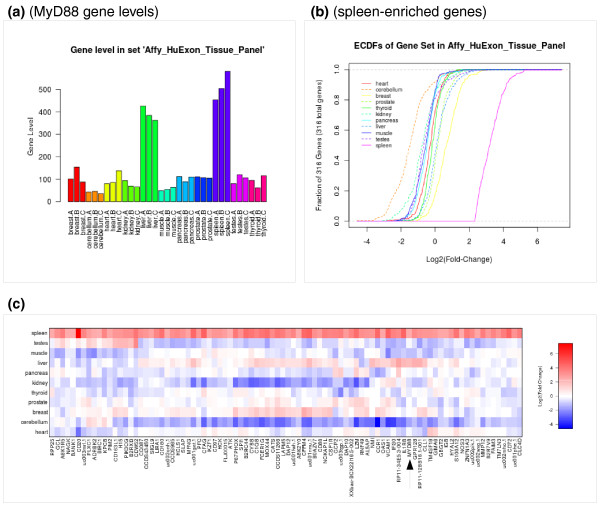
**ExpressionPlot screen shots examining spleen-enriched genes in human exon array tissue panel data **[28]. **(a) **Levels of Myd88, a key signaling protein in the innate immune system [29], in human tissues using the genelev tool. **(b) **ecdf showing tissue enrichment (fold change relative to all other tissues) of the 316 genes least 5-fold enriched in the spleen at a *P-*value cutoff of 10^-4^. The sharp angle at 2.3 in the spleen curve indicates the 5-fold cutoff. The position of the cerebellum curve to the left of all the others may reflect the general depletion of immune cells, which is characteristic of the spleen, within the nervous system. **(c) **event_heatmap showing the fold enrichments of the 316 spleen-enriched genes in all 11 tissues in the panel. The screen shot was edited by removing many of the genes from the middle for formatting purposes and adding an arrow to indicate Myd88, which is part of a cluster of spleen-enriched genes also enriched in the liver. The depletion of the spleen-enriched genes in the cerebellum is evident by the excess blue color in the cerebellum row.

The genelev tool generates barplots of gene levels (RPKM) with error bars (Figure 6a). The ecdf tool allows the user to visualize the levels or fold changes of a set of genes by plotting the cumulative distribution of those genes' levels in the samples of a project or fold changes in the pairwise comparisons of a project (Figure 6b). Instead of looking at the distribution of the whole set, the event_heatmap tool visualizes the individual levels or fold-change of all the genes in the set as a heatmap (Figure 6c).

### Administrative tasks

ExpressionPlot has an access-management system that makes it easy for end users to share their data or release it publicly. New user accounts can be made automatically through the website, including an e-mail-based password recovery feature. When invoking the back end for a given project one user is assigned 'admin' privileges. Users can then assign either 'view' or 'admin' privileges to other users on projects for which they are 'admin', or can add a 'public' flag to the project to make it visible without login. These permissions are all controlled via a simple web interface.

## Download, installation, help

Visit the ExpressionPlot website at [22] for instructions on how to download and install the latest version. ExpressionPlot requires an existing MySQL and Apache web server, as well as the RApache module. The install.pl script checks all the dependencies and tries to satisfy or make suggestions on how to satisfy any that are missing. It then downloads and installs the latest version of ExpressionPlot. Alternatively, a VirtualBox hard drive is available running Ubuntu linux with ExpressionPlot already installed. In either case, after installation is complete the EP-manage.pl script can be used to download and add on bowtie indexes, annotations and microarray library files as required. Example data sets, both unprocessed and processed, can also be installed using the same script. The User's Guide can be found at [23] and contains detailed instructions on setting up and running ExpressionPlot.

Please use the ExpressionPlot discussion group to post technical questions or hints. This can be accessed by visiting the ExpressionPlot Google group [24] or by sending e-mail to expressionplot@googlegroups.com.

## Extracting biological meaning from high throughput data

ExpressionPlot offers the gene expression community an easy-to-use tool for automated analysis of gene expression and RNA processing data. The back end offers a solution to the problem of detecting significant changes in gene expression and RNA processing, while the web-based interface offers data analysis, visualization and browsing tools that realize the biological potential of this new technology.

## Methods

### Calculating *P*-values for significance of changes in gene expression

Given total numbers of reads in two samples (or two groups of samples) *n*_1 _and *n*_2_, *g*_1 _and *g*_2 _of which align to a particular gene of interest, we model *g*_2 _as a binomial distribution with parameters *q*_2 _and g, where *q*_2 _= *n*_2_/(*n*_1 _+ *n*_2_), and *g *= *g*_1 _+ *g*_2 _is the total number of reads aligning to the gene in either sample. The (two-tailed) *P-*value is then calculated using R's binom.test() function.

### Minimize significant changes method to estimate effective total read numbers

To estimate the effective total number of reads *n*_1 _and *n*_2 _in a pair of samples (or pair of groups of samples), we estimate *q*_2_, which is the fraction of reads in the second sample, and then set *n*_2 _= *q*_2_*N *and *n*_1 _*= N *- *n_2 _*where *N *is the total number of uniquely aligning reads from either sample.

The theory of our calculation of *q*_2 _is that once a *P-*value cutoff is set, any potential choice of *q*_2 _will lead to a certain number of significantly changed genes, say *C*(*q*_2_), which could be calculated by applying the procedure described above to every gene (for example 27,389 genes in mouse). Thus, we have the optimization problem:

$$\underset{q_{2}}{\min}\;C\left(q_{2}\right):0\leq q_{2}\leq 1$$

Solving the problem by convex optimization methods would be feasible but slow due to the cost of re-calculating *C*(*q*_2_). Instead, we use the binconf() function from R's Hmisc library [25] to calculate a 95% confidence interval for *q*_2 _for every gene, based on the observed number of reads. This interval corresponds to the range of *q*_2 _for which that gene is not significantly changed. Then the range 0 to 1 is split into windows of width 0.0001, and the number of genes whose confidence interval overlaps each of these windows is counted. The uncertainty introduced by using windows as point estimates is mitigated by their small radius: a difference of 0.0001 (0.01%) in the sample size estimate will have a minute effect on resultant gene levels. The value of *q*_2 _for the window overlapped by the confidence intervals of the most genes (or the mean of the *q*_2 _for the several windows if there is a tie for the most intervals) is then taken as the optimum. Empirical tests show that this method is extremely robust to the choice of *P*-value cutoff (data not shown). This is implemented in a very short R function called minimize.significant.changes() in BradStats.R [26].

### European Nucleotide Archive accession numbers

The previously unpublished (and de-identified) data sets used to create Figure 2d, and Figures S7 and S9 in Additional file 1 are available from the European Nucleotide Archive under accession number ERP000619, available at [27].

### Archival copy of software

For archival purposes, version 1.3 of the software is included as Additional file 4, but it is recommended to use the latest version available through the website.

## Abbreviations

ECDF: empirical cumulative distribution function; PAC: poly-adenylation/cleavage site; RPKM: reads per kilobase gene model per million total reads; TSS: transcription start site.

## Competing interests

The authors declare that they have no competing interests.

## Authors' contributions

BF conceived of and wrote the software and the manuscript. TM helped in its design and coordination and in drafting the manuscript. Both authors read and approved the final manuscript.

## Supplementary Material

Additional file 1 **Supplementary figures, methods, references, and description of other additional files**. Click here for file

Additional file 2 **Data for Figure S7 in Additional file **1. Click here for file

Additional file 3 **Data for Figure **2d. Click here for file

Additional file 4 **Archival copy of software**. Click here for file

## References

[B1] Wang ET Sandberg R Luo S Khrebtukova I Zhang L Mayr C Kingsmore SF Schroth GP Burge CB Alternative isoform regulation in human tissue transcriptomes. Nature 2008 456 470 476 10.1038/nature07509 18978772 PMC2593745

[B2] Nagalakshmi U Waern K Snyder M RNA-Seq: a method for comprehensive transcriptome analysis. Curr Protoc Mol Biol 2010 Chapter 4 Unit 4.11.1-13 10.1002/0471142727.mb0411s89 20069539

[B3] Mortazavi A Williams BA McCue K Schaeffer L Wold B Mapping and quantifying mammalian transcriptomes by RNA-Seq. Nat Methods 2008 5 621 628 10.1038/nmeth.1226 18516045 PMC13303166

[B4] Trapnell C Pachter L Salzberg SL TopHat: discovering splice junctions with RNA-Seq. Bioinformatics 2009 25 1105 1110 10.1093/bioinformatics/btp120 19289445 PMC2672628

[B5] Trapnell C Williams BA Pertea G Mortazavi A Kwan G van Baren MJ Salzberg SL Wold BJ Pachter L Transcript assembly and quantification by RNA-Seq reveals unannotated transcripts and isoform switching during cell differentiation. Nat Biotechnol 2010 28 511 515 10.1038/nbt.1621 20436464 PMC3146043

[B6] Li H Ruan J Durbin R Mapping short DNA sequencing reads and calling variants using mapping quality scores. Genome Res 2008 18 1851 18581 10.1101/gr.078212.108 18714091 PMC2577856

[B7] Katz Y Wang ET Airoldi EM Burge CB Analysis and design of RNA sequencing experiments for identifying isoform regulation. Nat Methods 2010 7 1009 1015 10.1038/nmeth.1528 21057496 PMC3037023

[B8] Robinson JT Thorvaldsdóttir H Winckler W Guttman M Lander ES Getz G Mesirov JP Integrative Genomics Viewer. Nat Biotechnol 2011 29 24 26 10.1038/nbt.1754 21221095 PMC3346182

[B9] Langmead B Trapnell C Pop M Salzberg SL Ultrafast and memory-efficient alignment of short DNA sequences to the human genome. Genome Biol 2009 10 R25 10.1186/gb-2009-10-3-r25 19261174 PMC2690996

[B10] Wu Z Jenkins B Rynearson T Dyhrman S Saito M Mercier M Whitney L Empirical bayes analysis of sequencing-based transcriptional profiling without replicates. BMC Bioinformatics 2010 11 564 10.1186/1471-2105-11-564 21080965 PMC3098101

[B11] Goecks J Nekrutenko A Taylor J Galaxy: a comprehensive approach for supporting accessible, reproducible, and transparent computational research in the life sciences. Genome Biol 2010 11 R86 10.1186/gb-2010-11-8-r86 20738864 PMC2945788

[B12] Reich M Liefeld T Gould J Lerner J Tamayo P Mesirov JP GenePattern 2.0. Nat Genet 2006 38 500 501 10.1038/ng0506-500 16642009

[B13] Fujita PA Rhead B Zweig AS Hinrichs AS Karolchik D Cline MS Goldman M Barber GP Clawson H Coelho A Diekhans M Dreszer TR Giardine BM Harte RA Hillman-Jackson J Hsu F Kirkup V Kuhn RM Learned K Li CH Meyer LR Pohl A Raney BJ Rosenbloom KR Smith KE Haussler D Kent WJ The UCSC Genome Browser database: update 2011. Nucleic Acids Res 2011 39 Database D876 882 20959295 10.1093/nar/gkq963 PMC3242726

[B14] Hubbard TJP Aken BL Ayling S Ballester B Beal K Bragin E Brent S Chen Y Clapham P Clarke L Coates G Fairley S Fitzgerald S Fernandez-Banet J Gordon L Graf S Haider S Hammond M Holland R Howe K Jenkinson A Johnson N Kahari A Keefe D Keenan S Kinsella R Kokocinski F Kulesha E Lawson D Longden I Ensembl 2009. Nucleic Acids Res 2009 37 D690 697 10.1093/nar/gkn828 19033362 PMC2686571

[B15] Anders S Huber W Differential expression analysis for sequence count data. Genome Biol 2010 11 R106 10.1186/gb-2010-11-10-r106 20979621 PMC3218662

[B16] Marioni JC Mason CE Mane SM Stephens M Gilad Y RNA-seq: an assessment of technical reproducibility and comparison with gene expression arrays. Genome Res 2008 18 1509 1517 10.1101/gr.079558.108 18550803 PMC2527709

[B17] Robinson MD Oshlack A A scaling normalization method for differential expression analysis of RNA-seq data. Genome Biol 2010 11 R25 10.1186/gb-2010-11-3-r25 20196867 PMC2864565

[B18] Bullard J Purdom E Hansen K Dudoit S Evaluation of statistical methods for normalization and differential expression in mRNA-Seq experiments. BMC Bioinformatics 2010 11 94 10.1186/1471-2105-11-94 20167110 PMC2838869

[B19] Affymetrix - Affymetrix Power Tools. http://www.affymetrix.com/partners_programs/programs/developer/tools/powertools.affx

[B20] Smyth GK Linear models and empirical bayes methods for assessing differential expression in microarray experiments. Stat Appl Genet Mol Biol 2004 3 Article3 10.2202/1544-6115.1027 16646809

[B21] Oshlack A Wakefield M Transcript length bias in RNA-seq data confounds systems biology. Biol Direct 2009 4 14 10.1186/1745-6150-4-14 19371405 PMC2678084

[B22] ExpressionPlot. http://expressionplot.com/

[B23] ExpressionPlot User's Guide. http://expressionplot.com/wiki

[B24] ExpressionPlot Google Group. http://groups.google.com/group/expressionplot

[B25] CRAN - Package Hmisc. http://cran.r-project.org/web/packages/Hmisc/index.html

[B26] BradStats.R - expressionplot - Project Hosting on Google Code. http://code.google.com/p/expressionplot/source/browse/trunk/lib/R/BradStats.R

[B27] European Nucleotide Archive: ERP000619. http://www.ebi.ac.uk/ena/data/view/ERP000619

[B28] Affymetrix - Sample Data, Exon 1.0 ST Array Dataset. http://www.affymetrix.com/support/technical/sample_data/exon_array_data.affx

[B29] Akira S Takeda K Toll-like receptor signalling. Nat Rev Immunol 2004 4 499 511 10.1038/nri1391 15229469

