# Supplementary material for: ExpressionPlot: a web-based framework for analysis of RNA-Seq and microarray gene expression data
Source: Genome Biol. 2011 Jul 28;12(7):R69. doi: 10.1186/gb-2011-12-7-r69 (PMC3218831; doi:10.1186/gb-2011-12-7-r69)
Supplement: Additional file 1 — Supplementary figures, methods, references, and description of other additional files. [file gb-2011-12-7-r69-S1.PDF]

Supplementary Information File 1:

- **Supplementary Figures and Legends**
- **Supplementary Methods**
- **List of Additional Files**
- **References for Supplementary Figure Legends and Methods**

Supplementary Figures

Supplementary Figure 1. Single-end alignment flowchart.

Single end reads are aligned to the genome with bowtie using the arguments “-k 2 -m 200 --best --strata”. Only best hits are considered. Multiple-best-aligning reads are discarded (right branch). Non-aligning reads are then aligned to the junction database using bowtie with the same options. Now multiple-best-aligning reads as well as non-aligning reads are discarded. Unique best genome and junction alignments are then further processed by the pipeline for gene expression analysis.

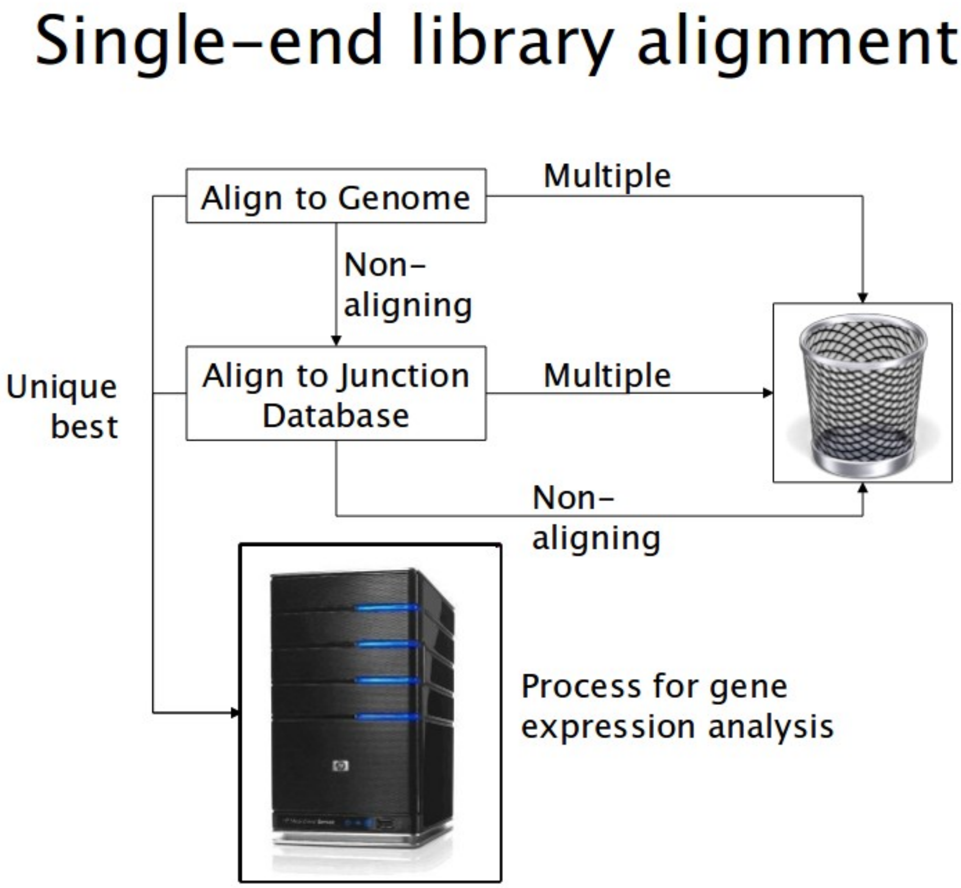

Supplementary Figure 2. Paired-end alignment flowchart.

Paired end reads are aligned to the genome using bowtie with arguments “-X 20000 -k 2 -m 2”. Multiple-aligning reads are discarded. Single-ends of non-pair-aligning reads are then again aligned to the genome with bowtie (arguments “-k 2 -m 200 --best --strata”) and multiple-aligning reads are discarded. Non-aligning reads from this step are then aligned to the junction database using the same arguments. Finally multiple-aligning as well as non-aligning reads are discarded.

The unique best single-end alignments from the genome and junction steps are then re-paired. These pairs are combined with the genomic paired-end alignments from the first step, then, along with the single-end alignments that could not be re-paired, are sent downstream for further gene expression analysis processing.

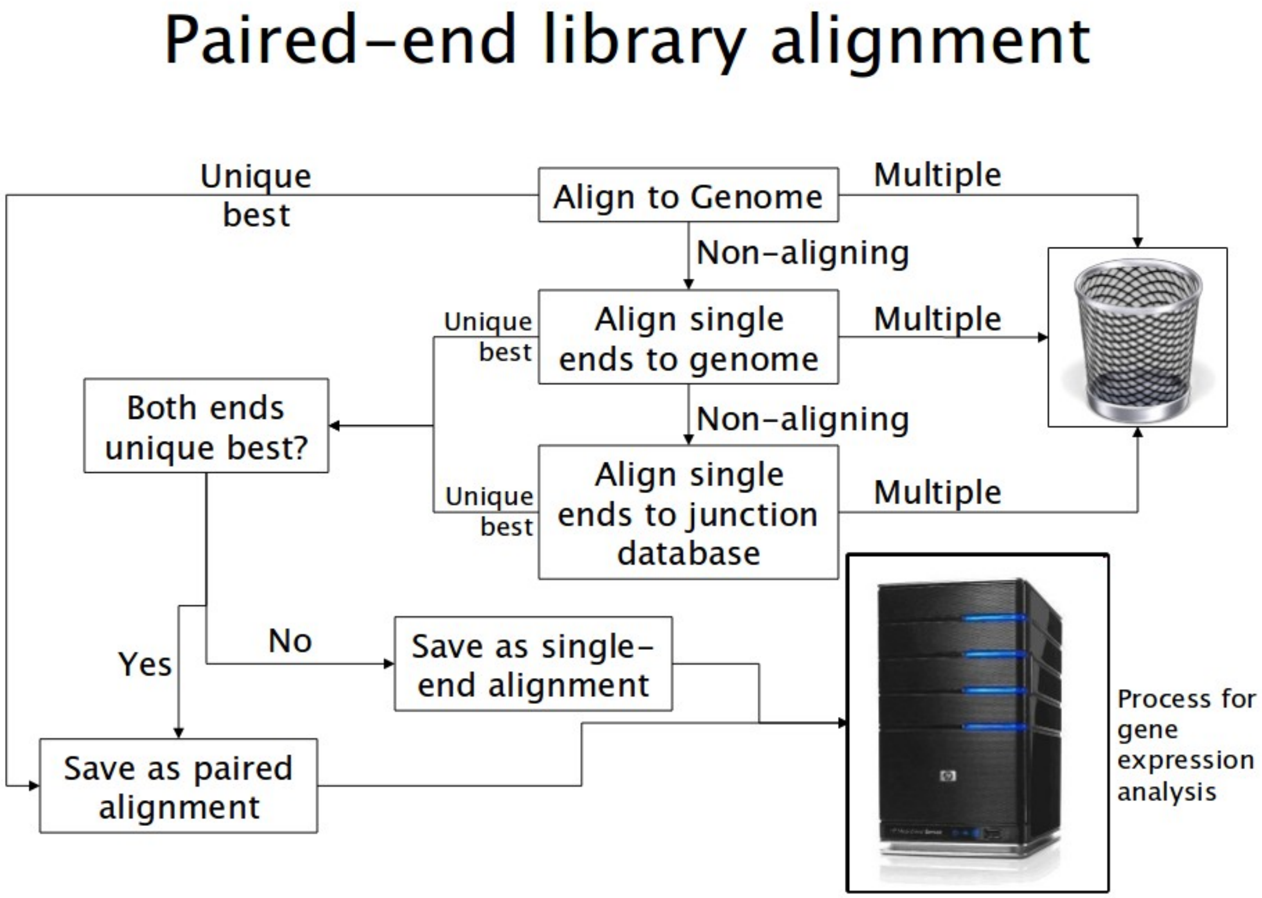

Supplementary Figure 3. Quantifying cassette exon usage.

Cassette exons are quantified by comparing inclusion reads to skipping reads. Inclusion reads include junction reads ending or beginning at either splice site of that exon as well as exon body reads. Skipping reads are junction reads that flank the candidate exon but are anchored in known splice sites of the host gene. The main reason for the anchor requirement is to prevent the “skipping reads” count for exons of intron-encoded genes from including junction reads of their host introns. For paired-end data reads are counted as inclusion or skipping if either mate is a skipping or inclusion alignment.

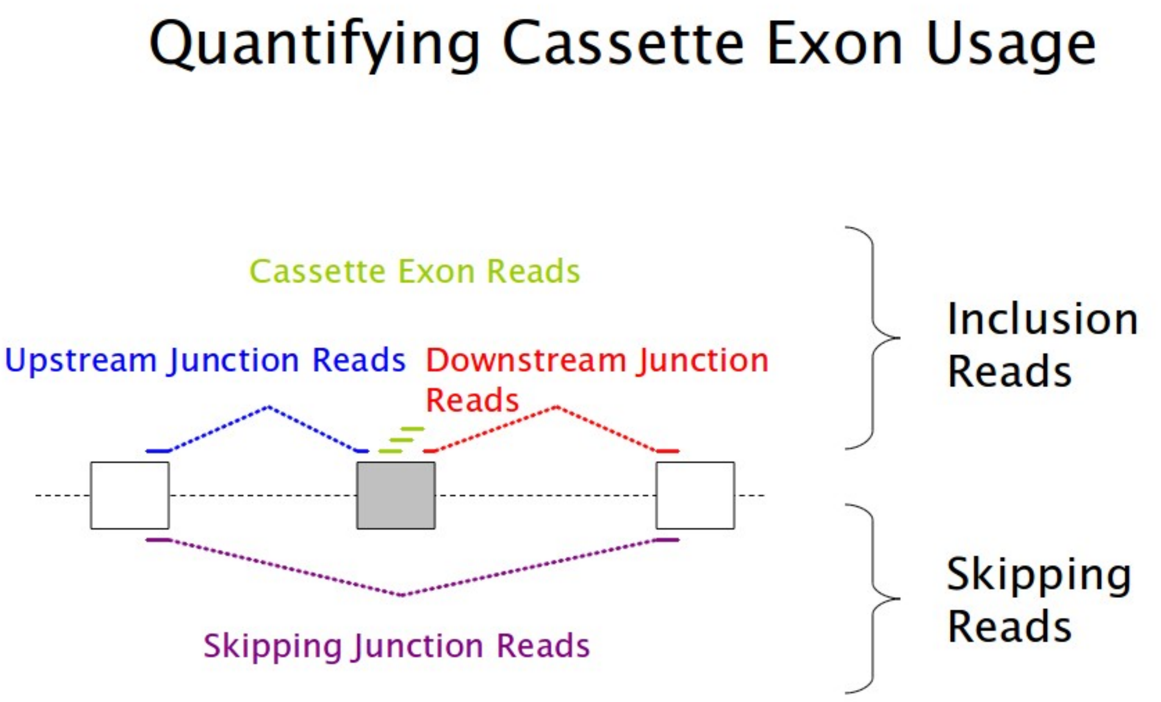

Supplementary Figure 4. Quantifying intron retention.

For intron retention, the number of reads aligning to the intron is compared to the number aligned to flanking exons (actually to the “locally constitutive” transcript---see Supplementary Figure 5). The number of splice junction reads is counted but not considered for statistical purposes. As a default, only introns with a read density (RPKM) at least 2.5% of the flanking regions are considered.

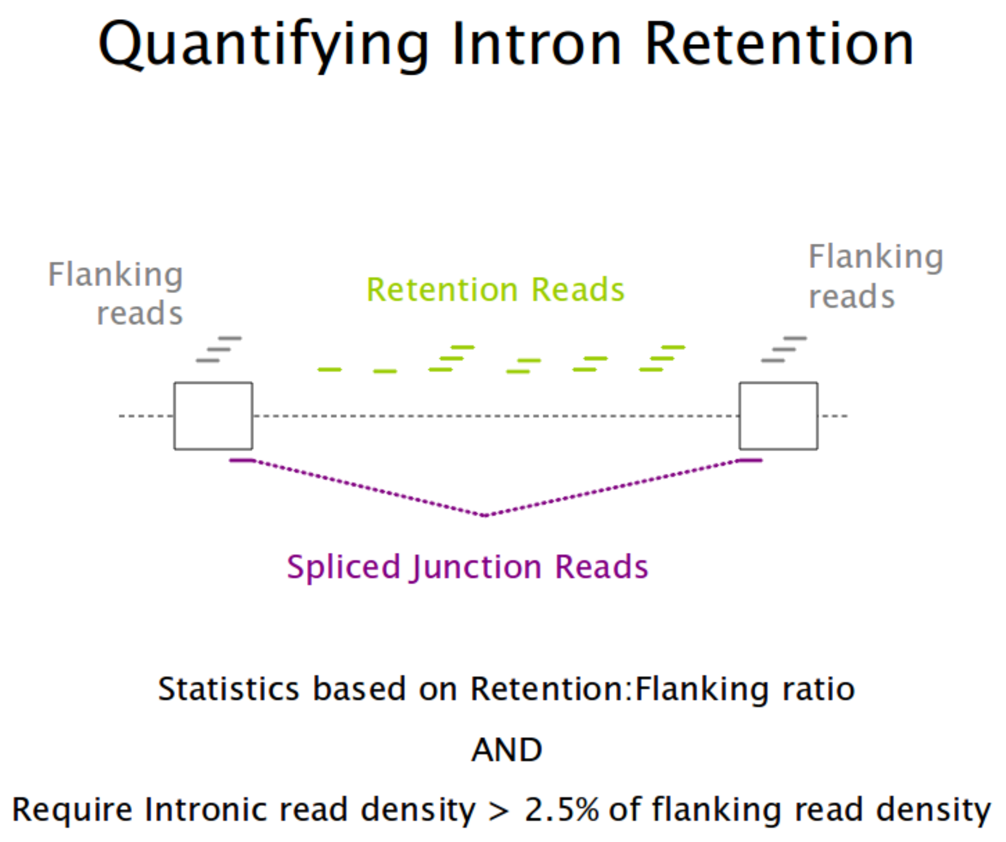

The 2.5% figure can be adjusted by editing line 664 of `expressionplot-config`/lib/R/ExpressionPlot.R:

```
RNASeq.RetainedIntron.P <- function(tbl, ifr.min=0.025)  {
```

`ifr.min` stands for “intron-flank ratio minimum”. Change `ifr.min=0.025` to the desired amount, or set to 0 to remove filter entirely.

Supplementary Figure 5. Example of “locally constitutive” exons for a candidate intron.

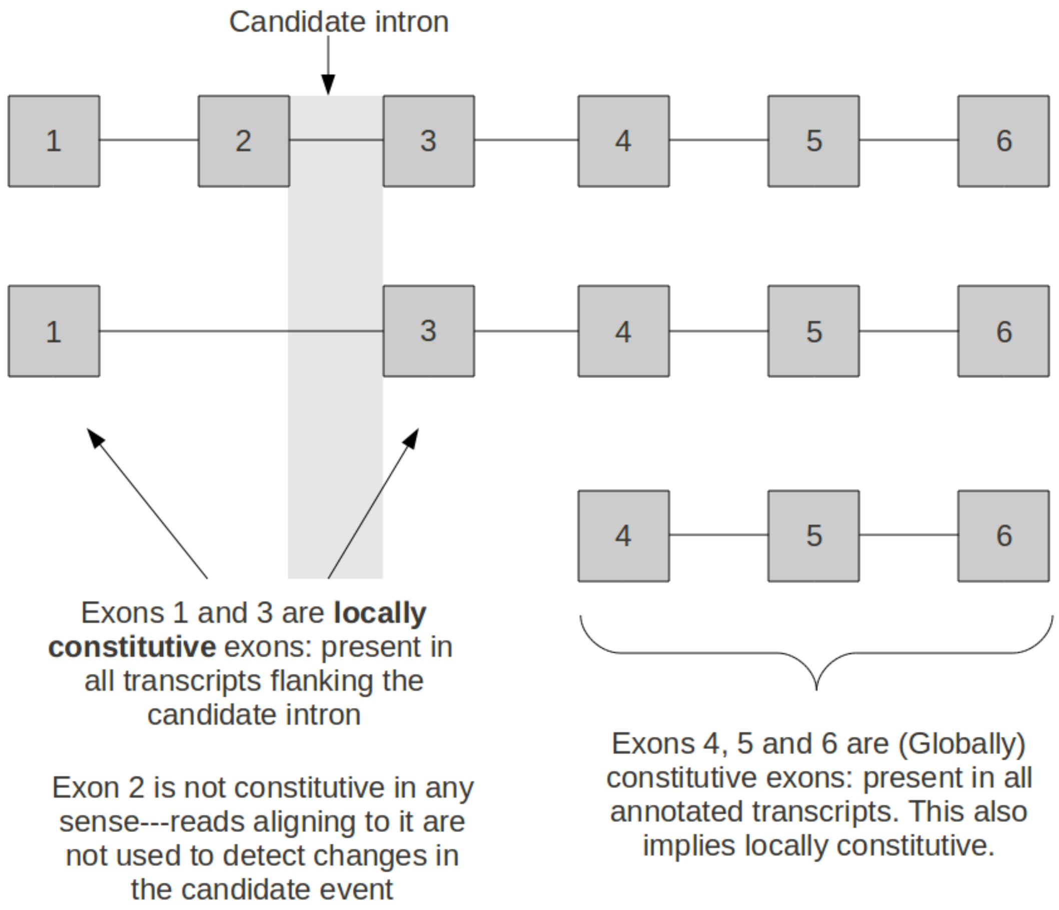

Supplementary Figure 6. Quantifying terminal exon usage.

Alternative terminal exons (ATEs) are quantified by comparing proximal reads to distal reads. This figure shows as an example the 3' end, but the algorithm is identical for both 5' and 3' ends. For each gene, all transcript termini but the most distal are considered as alternative terminal exon events. (These events are really testing reads associated with the termini, and not with, for example, the splice sites of the terminal exons). Each terminus is categorized as “intronic” if it is within the intron of a more distally terminating transcript (top and bottom left) and “exonic” if in the exon of a more distally terminating transcript (bottom right). All termini are exonic or intronic, and it is also possible to be both (not shown). Distal reads include genomic reads that align to distal exon bodies or, in the case of intronic termini, junction reads that splice over the terminus but are anchored in known splice sites of the gene. Proximal reads for intronic termini are those that align to the terminal exon but are downstream of the last 5' splice site (for 3' termini; upstream of the first 5' splice site for 5' termini) proximal to the terminus. For purely exonic termini the entire exon through to the terminus is used.

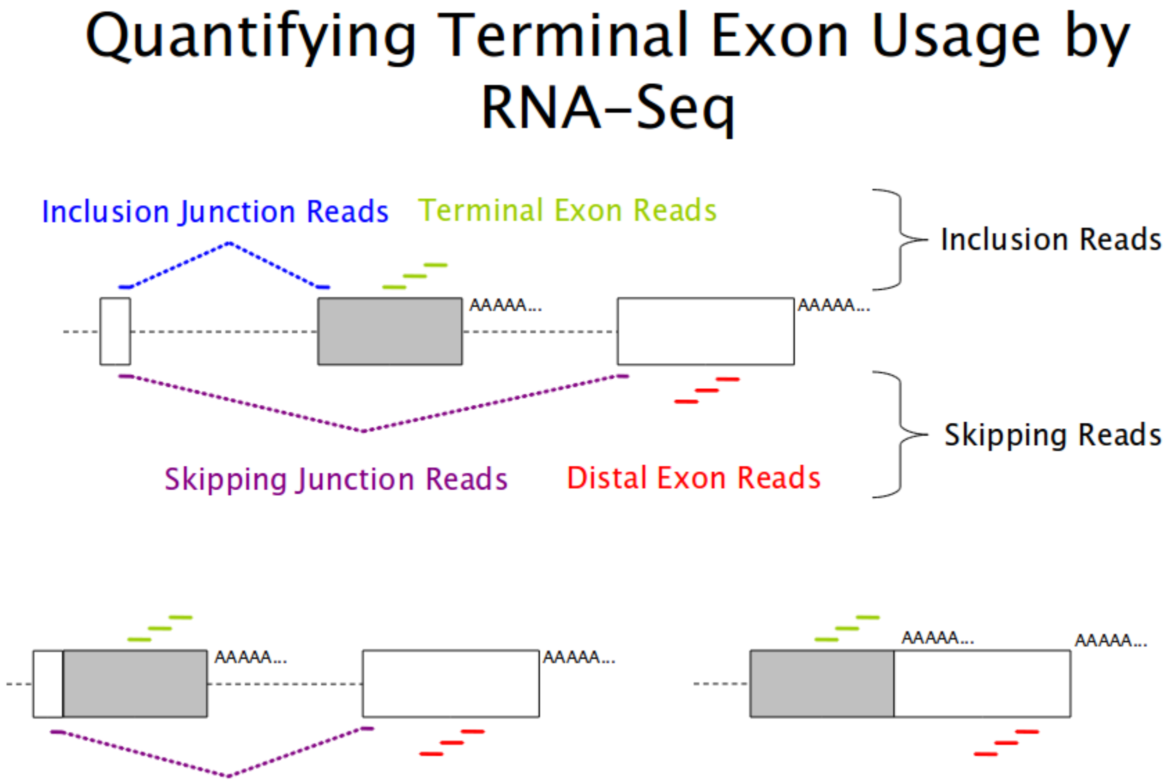

Supplementary Figure 7. Comparison of DESeq and MSC methods.

DESeq and MSC/binomial test methods were run on two contrasts (data in Additional File 2). The first (1x1, left column) compared gene expression from wildtype experiment to one mutant experiment (one lane of RNA-Seq each). The second (2x2, right column) compared gene expression from the same experiment as well as a duplicate (a wildtype and mutant lane from cells prepared on 2 different days). DESeq keeps the duplicates separate and models the population variation whereas MSC/binomial test pools the duplicates and models only technical variation.

The first two rows show the calculated RPKMs for the wildtype and mutant samples. MSC-calculated RPKM is on the *x*-axis and DESeq on the *y*-axis. There is near-identical agreement in these values (dashed gray line indicates *y*=*x*).

The third row shows the calculated log2-fold-changes from the two methods. Here there is near-identical agreement again, except that MSC tends to shift the log2-fold-changes slightly towards 0. The reason for this more conservative approach is that 2 pseudocounts are added before calculating the fold changes (this has no effect on the statistical calculations of the P-values).

The fourth row compares the *P*-values from the two methods. Here there are significant differences, with MSC/binomial usually much more significant that DESeq. The red box counts genes in the extreme cases of $P_{DESeq} \geq 0.05$ and $P_{MSC} \leq 10^{-4}$. The numbers of such genes, regardless of the size of the fold change, are shown---note that this number is decreased in the 2x2 case where DESeq is more powerful. These represent genes technically significantly different but not biologically significantly different.

The last looks at the RPKM values of genes in wildtype and mutant cells and colors those “boxed” genes from the fourth row green if they have a fold-change of at least 2 up or down, and red otherwise. The number of genes in each category is also shown in the corners. Adding this 2-fold filter shows that in fact there are no “technical false positives” in the 2x2 set.

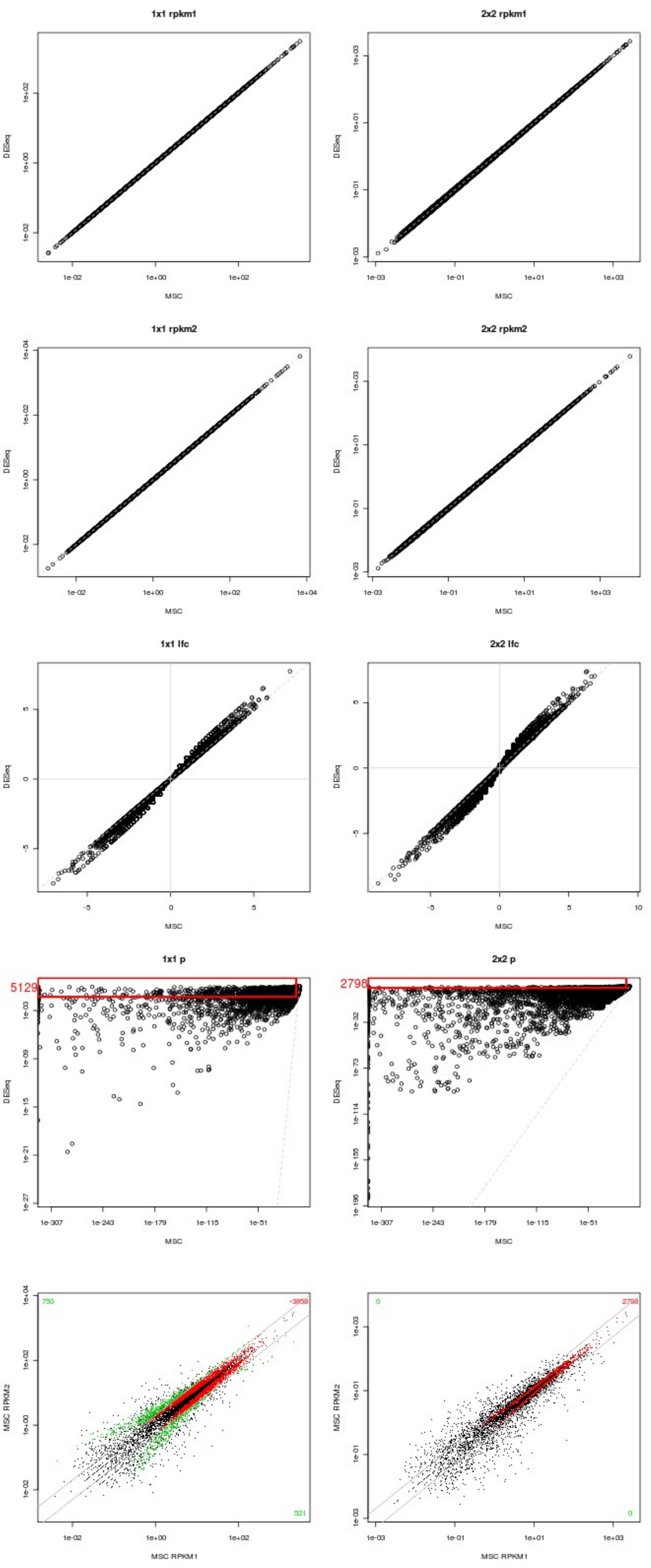

Supplementary Figure 8. Example of **heatmap** plot from *ExpressionPlot.*

Example plots from *ExpressionPlot* comparing tissue-enriched gene expression in human exon array tissue panel data[1] (*x*-axis) and human RNA-Seq tissue panel data[2] (*y*-axis). For each pair of tissues (t_e, t_r), where t_e is a tissue from the exon array panel and t_r is a tissue from the RNA-Seq panel, a 2x2 contingency table was constructed counting the number of genes significantly changed up and down in both tissues and data sets. Fisher's exact test or the chi-squared test was then applied and the *P*-value was then colored into the square (t_e, t_r). Dots indicate anti-correlation. Red colors are not significant, and significance increases through yellow and white. Notable positive correlations include (cerebellum, brain), (breast, breast), (liver, liver), (heart, heart), (muscle, skel_muscle), (breast, adipose), (testes, testes) and (spleen, lymph_node). None of the exon array tissues correlate well with colon. Adipose RNA-Seq correlates better than breast RNA-Seq with exon array breast by this measure but this may be due to deeper sequencing.

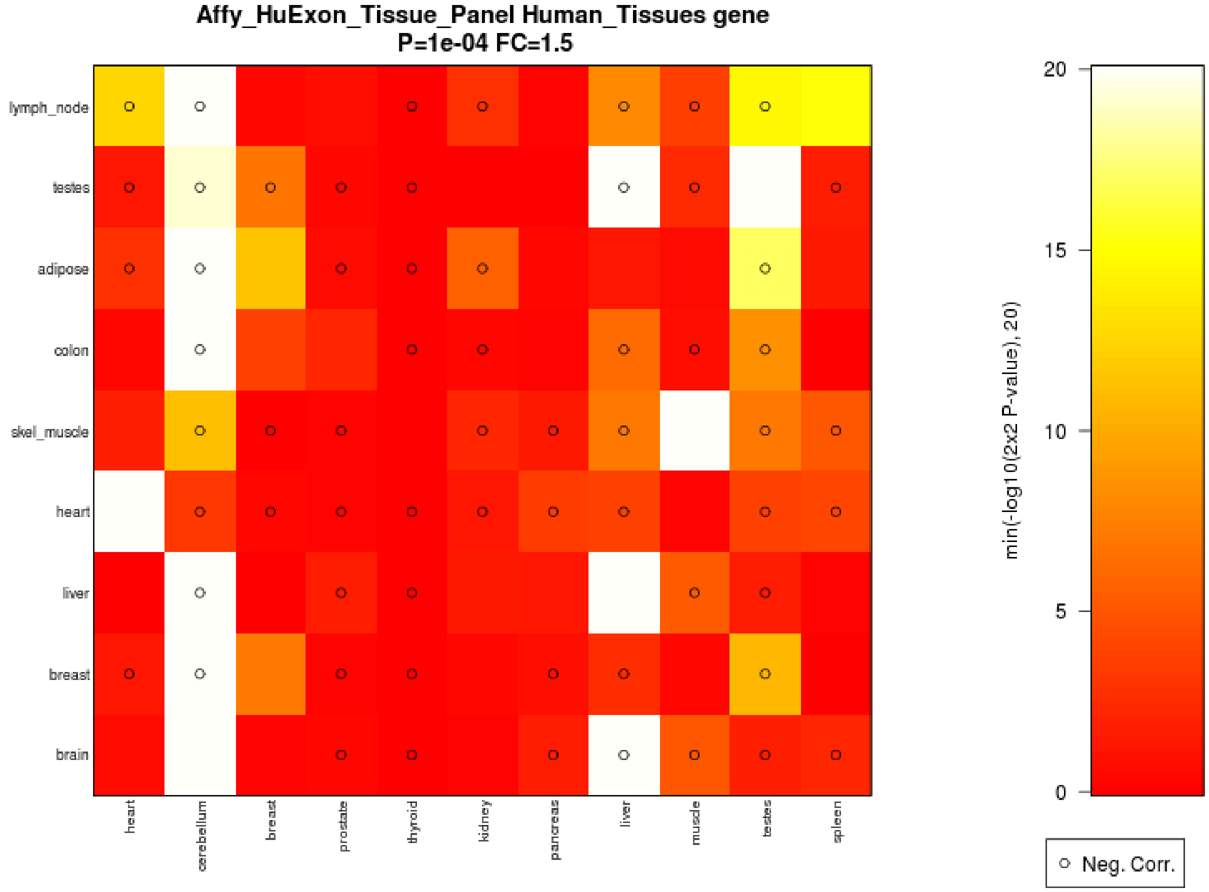

Supplementary Figure 9. Example of **pairplot** plot from *ExpressionPlot.*

This pairplot shows the vicinity of an alternatively spliced exon in Clta (clathrin light chain A) in a paired-end data set. Bottom track shows known transcripts in this region. Second track from bottom shows read pileup for this sample throughout the region. Top section shows the “pairplot”. At position (*x*, *y*), where *x* and *y* are coordinates within the chromosomal window, a point is plotted if there is a paired-end read whose plus-strand aligned read has its last base aligned to *x* and its minus-strand aligned read with its last base aligned to *y* (reads aligning to different chromosomes, same strand, or with *x* > *y* - *readlen* are not included in this plot, but counted and barplotted by the **pairdist** tool online). The color and weight of the point indicate the number of such reads. Multiple *y* values for a single *x* value indicate alternative splicing. For example, there are a few small red dots above the first exon corresponding to pairs spanning the first and second exon. The larger cyan dot above indicates reads spanning the first and fourth (last in this window) exon, and, concordant with the pileup plots, indicate that the skipping isoform is more abundant. Above the second exon one can also see a few reads going to the third as well as the fourth. Thus the double-inclusion isoform (1-2-3-4) is also present, but at an ever lower level than the single inclusion 1-2-4 isoform. No paired-end reads support a 1-3 splice, so the 1-3-4 isoform, if it exists at all, is scarce. This particular data set had an insert size close to twice the read length. This is why most of the reads lie along the gray diagonal *y*=*x.* (The underlying data set has been de-identified, and is available upon request).

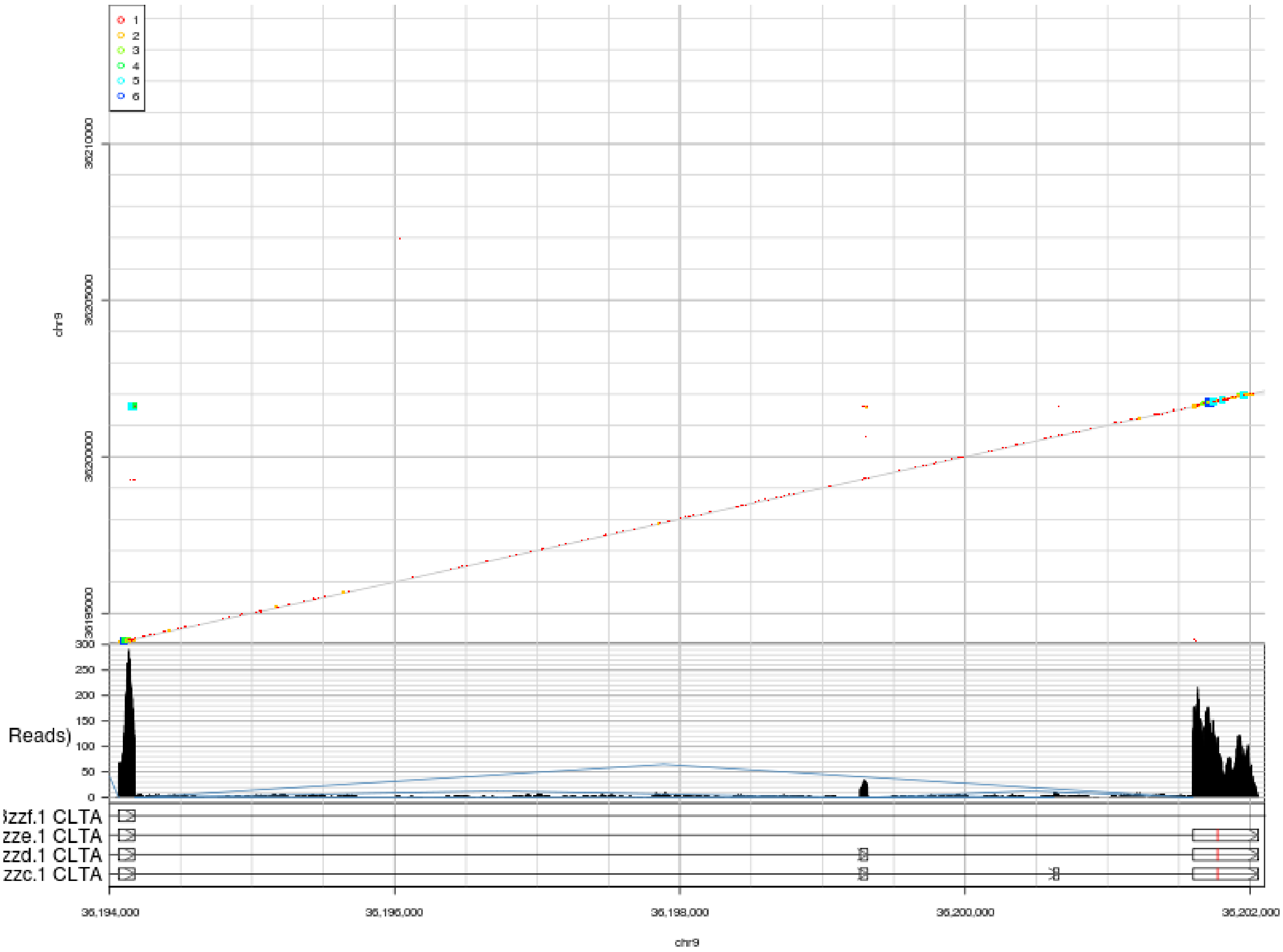

Supplementary Figure 10. Fisher's Exact Test is Conservative for Isoform Ratio Tests.

These plots show the cumulative distribution of Fisher's P-values (on a log scale), with the line *y*=*x* overplotted in gray. The position of the points below that line indicate that the *P* values are actually conservative, since the probability of getting a particular *P*-value or smaller (*y*-axis) is actually less than the *P*-value itself. This discrepancy disappears with larger *n.*

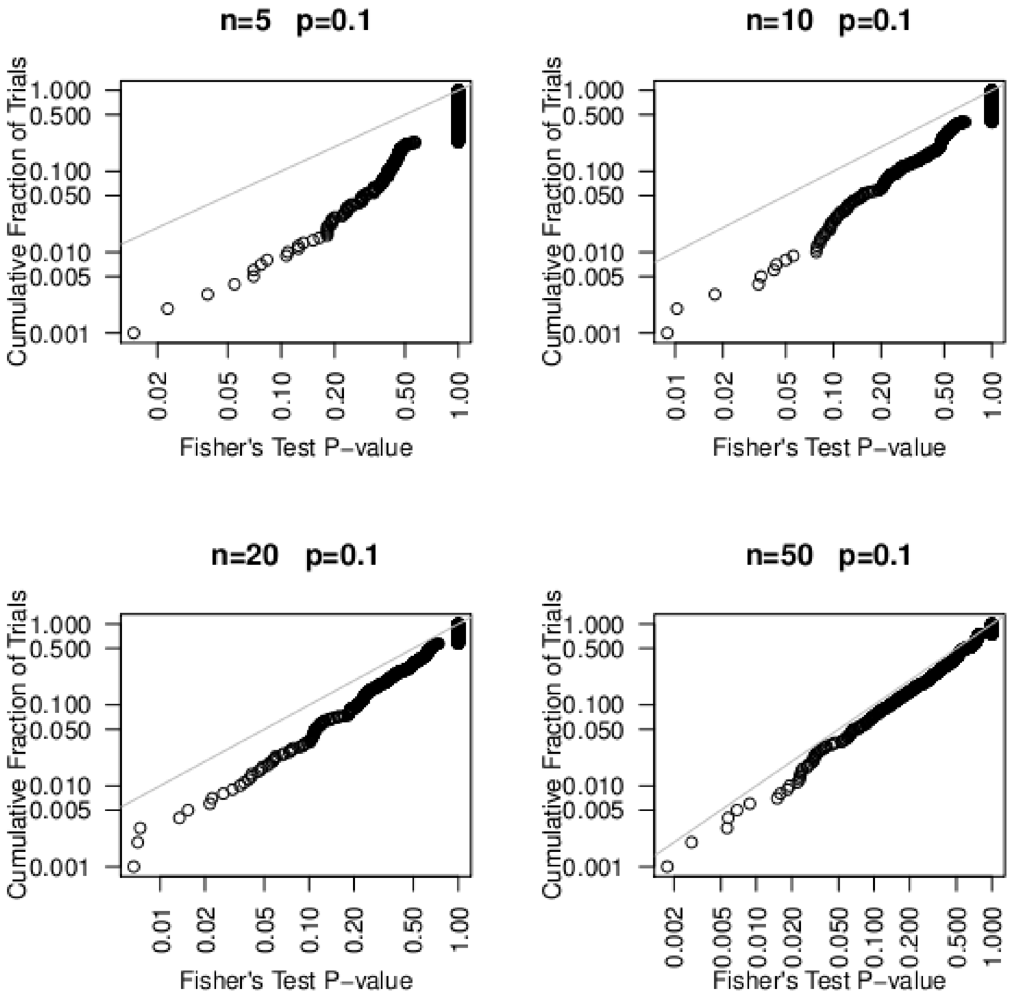

Supplementary Methods

Correlation Tool

The correlation tool begins with the log-transformed levels (median probe intensities or RPKMs) for every gene in every sample of a project. Pairwise (pearson) correlations are then calculated between every pair of samples. These correlations are then displayed as a matrix (in “heatmap” mode) or a clustering dendrogram (based on 1-correlation) generated by R's hclust() function with default values.

Heatmap Tool

A reliable way to compare transcriptional profiles from disparate samples and platforms is to focus on changes rather than absolute levels, because each comparison is then well controlled. While the 4way tool is the best for comparing two change profiles, This tool allows you to visualize many such comparisons at once, represented as a grid of colored squares. The *x*-axis corresponds to a project, each column associated with a different comparison of that project, and the *y*-axis corresponds to a (possibly different) project, each row associated with a different comparison of that project. The *x*-axis comparisons are related to the *y*-axis comparisons ("all-versus-all"), and the grid squares are colored to indicate the extent of the correlation or anti-correlation of the two change profiles. In this way each square of the heatmap summarizes a 4-way plot. The user may choose from one of several different methods to calculate a numerical value for the associations. See the User's Guide for details on these methods: http://www.expressionplot.com/wiki/index.php?title=Heatmap.

Isoform Comparisons

For each class of RNA processing event analyzed (cassette exon, alternative terminal exon, retained intron), reads supporting either the candidate region (which could be an exon, part of an exon, or an intron) or the alternative to the candidate region (e.g. skipping isoform for an exon, or spliced isoform for an intron) are counted in both samples. The definitions of the different read classes for each event type is given in Figs. S3, S4 and S6. A 2x2 contingency table is then formed for each candidate event. For example, for cassette exons the table looks like this:

	Sample 1	Sample 2
Inclusion	Number of inclusion reads for sample 1	Number of inclusion reads for sample 2
Skipping	Number of skipping reads for sample 1	Number of skipping reads for sample 2

Fisher's test or a Chi-Squared test is then used to ask if the ratio of inclusion to skipping reads is significantly different in the two samples. This is similar previously described methods[2].

Comparison of Differential Expression Methods

We found that for data sets with changes in the expression levels of the most highly expressed genes the naïve method of using the total number of aligning reads leads to biases. (This has since been noted several times in the literature.) We therefore developed a procedure we call *Minimize Significant Changes* (MSC). To our knowledge this is a new way of normalizing RNA-Seq data (although we expect that the results are similar to those generated by previously described methods). A mathematically equivalent way to describe this method is that each gene “votes” on a confidence

interval for the scaling factor between two samples, and the point overlapping the most confidence intervals is chosen as the scaling factor. In most experiments these effective total read numbers are similar to those derived from using total number of aligning reads, but when the most highly expressed genes are changed this procedure is more effective at controlling for the levels of most genes.

Recently, another method called *Scaling Normalization* was presented[5] in which the scaling factor is calculated as a precision-weighted trimmed mean of the log-fold-changes in the total raw reads for each gene (so-called *M*-values). Since the weighting is based on the precision (inverse variance), both methods give more weight to genes with more reads, and therefore would likely give very similar results. The DESeq package[4], on the other hand, uses the median fold-change to normalize RNA-Seq profiles. This method would also be robust to changes in highly expressed genes.

Comparing the differences in normalization and statistical approaches between our method (MSC/binomial test) and that of DESeq, we found that the normalizations yielded essentially identical values for gene expression levels and fold-changes (Figure S7 and Additional File 2). As expected, the binomial test gave much smaller (more significant) *P*-values. However, most of the genes called as significantly different by the binomial test but not by DESeq had very small fold change (less than 2). Interestingly, in a comparison with no replicates (one wildtype lane versus one mutant lane), we found one very highly expressed gene (Fn1) which showed a 5-fold reduction in RPKM from ~530 (represented by 117,000 reads) to 93 (represented by 22,000 reads), yet the *P*-value generated by DESeq was only 0.53. By contrast, the binomial test gave a significant *P*-value (10^{-323}). This suggests that for the special case of data sets with unreplicated samples it might be useful to perform technical comparisons rather than leaning on the assumptions inherent in modeling unknown variation in the DESeq or similar methods. We leave this decision up to the user.

Fisher's Exact Test is Conservative for Isoform Ratio Tests

Fisher's Exact Test is conservative because it fixes both margins. In reality, since each RNA-Seq is a random sample from a large pool of potential reads, the counts in the 2x2 contingency table can be thought of as independent Poisson distributions. To confirm that Fisher's Exact test is conservative we generated random 2x2 contingency tables according to the following probability distributions:

	Sample 1	Sample 2
Inclusion	Pois(*pn*)	Pois(*pn*)
Skipping	Pois((1-*p*)n)	Pois((1-*p*)*n*)

(*n* here is a parameter that we varied at different total number of counts, and *p* is a probability parameter that could be varied between 0 and 1, but for which we examined *p*=0.1) This represents a cassette exon event which has 9 times more skipping reads than inclusion reads. The following R code generates these tables:

```
m <- matrix(rpois(4, n*c(p,1-p)), nrow=2)
```

We then calculated there Fisher's exact P-value for each of the matrices over 1000 iterations (with `fisher.test(m)$p.value`) and plotted the distribution of P-values.

The plots in Figure S10 show the cumulative distribution of Fisher's P-values (on a log scale), with the line *y*=*x* overplotted in gray. The position of the points below that line indicate that the *P* values are actually conservative, since the probability of getting a particular *P*-value or smaller (*y*-axis) is actually less than the *P*-value itself. This discrepancy disappears with larger *n.*

Additional Files

Additional File 1: This document.

Additional File 2 (additional_file_2.zip): raw gene counts in one-on-one and two-on-two comparisons. RNA-Seq analysis was performed on two different preparations of wildtype and mutant cells (replicates 1 and 2). The data were analyzed in three ways, rep1 alone, rep2 alone, or both replicates together; by two different algorithms, MSC/binomial test and DESeq. The results of the analysis form the six files in the .zip archive:

combined.DESeq.tsv
combined.MSC.tsv
rep1.DESeq.tsv
rep1.MSC.tsv
rep2.DESeq.tsv
rep2.MSC.tsv

Each file is a tab-separated-values file. They can be opened in R, Excel, or OpenOffice. In R, for example, use read.table(“rep1.MSC.tsv”, header=TRUE, sep=”\t”, quote=””) to parse the files correctly.

The fields of the tables are as follows (Y/N indicates the presence of the given field in the files from that algorithm).

Field	MSC	DESeq	Description
clusterID	Y	Y	UCSC clusterID
aliases	Y	Y	Aliases for gene
ID	Y	Y	Repeat of clusterID field
n1	Y	Y	Number of reads in wildtype*
n2	Y	Y	Number of reads in mutant*
p	Y	Y	P-value for gene level change
lfc	Y	Y	Log2 fold-change
rpkm1	Y	Y	RPKM in wildtype
rpkm2	Y	Y	RPKM in mutant
id	N	Y	Repeat of clusterID field
baseMean	N	Y	DESeq only: mean of n1, n2
foldChange	N	Y	DESeq only: fold-change for gene level (linear)
padj	N	Y	DESeq only: B-H-adjusted P-value
resVarA	N	Y	DESeq only: Variance in wildtype
resVarB	N	Y	DESeq only: Variance in Mutant

* For MSC the “number of reads” is just that: the raw number of reads aligned to that gene model. However, for DESeq the “number of reads” is the normalized number of reads, rescaled by the sizeFactors.

Additional File 3 (additional_file_3.zip): Inner-mate distances from Figure 2D. The file “ecdf.tsv” within this archive gives the ECDFs plotted in figure 2D. The first column “d” gives the inner-mate distance, and the other columns (“A” through “H”) give the ECDF values for each of the 8 (de-identified) samples.

Additional File 4 (additional_file_4.zip): Software archive. This is a freeze of the software as of May 24, 2011. This is version 1.3 of *ExpressionPlot* and is included in the manuscript for review and archival purposes only. It is recommended to follow the installation directions on the website rather than those here—this will ensure that the latest version is installed.

To install anyway from the supplemental file first unzip it. You should find the following contents:

```
Archive:  SuppFile3.zip
  Length     Date   Time    Name
 -------     ----   ----    ----
    2770  12-17-10 07:25   expressionplot-install/install-config.ini
   51541  03-21-11 10:19   expressionplot-install/install.pl
 2895265  03-21-11 10:26   expressionplot-install/expressionplot-1.3.tar.gz
 -------                   -------
 2949576                   3 files
```

Enter the expressionplot-install directory and run install.pl, supplying the switch

```
-from-file expressionplot-1.3.tar.gz
```

to install.pl to use the file from the archive rather than downloading the current version. You will still need to check that Mysql, Apache and R are properly installed. It is handy also to create the MySQL user, password and database which ExpressionPlot will use, and supply those in install-config.ini. See the installation instructions for more information: http://www.expressionplot.com/wiki/index.php?title=Regular_Installation.

References for Supplemental File

1. **Affymetrix - Sample Data, Exon 1.0 ST Array Dataset** [http://www.affymetrix.com/support/technical/sample_data/exon_array_data.affx]

2. Wang ET, Sandberg R, Luo S, Khrebtukova I, Zhang L, Mayr C, Kingsmore SF, Schroth GP, Burge CB: **Alternative isoform regulation in human tissue transcriptomes**. *Nature* 2008, **456**:470-47610.1038/nature07509Available: Accessed 1 November 2010.

3. Bullard J, Purdom E, Hansen K, Dudoit S: **Evaluation of statistical methods for normalization and differential expression in mRNA-Seq experiments**. *BMC Bioinformatics* 2010, **11**:9410.1186/1471-2105-11-94Available: Accessed 23 May 2011.

4. Anders S, Huber W: **Differential expression analysis for sequence count data**. *Genome Biol* 2010, **11**:R10610.1186/gb-2010-11-10-r106Available: Accessed 21 November 2010.

5. Robinson MD, Oshlack A: **A scaling normalization method for differential expression analysis of RNA-seq data**. *Genome Biol* 2010, **11**:R2510.1186/gb-2010-11-3-r25Available: Accessed 21 November 2010.
