# Supplementary material for: ExpressionPlot: a web-based framework for analysis of RNA-Seq and microarray gene expression data
Source: Genome Biol. 2011 Jul 28;12(7):R69. doi: 10.1186/gb-2011-12-7-r69 (PMC3218831; doi:10.1186/gb-2011-12-7-r69)
Supplement: Additional file 4 — Archival copy of software. [file gb-2011-12-7-r69-S4.ZIP › expressionplot-install/expressionplot-1.3.tar.gz!/expressionplot-1.3/htdocs/index.html]

# ExpressionPlot

Good job! You installed ExpressionPlot!- ExpressionPlot on this Machine- ExpressionPlot home page
